# Commentary: The polarization-index: a simple calculation to distinguish polarized from non-polarized training intensity distributions

**DOI:** 10.3389/fphys.2023.1179769

**Published:** 2023-10-26

**Authors:** Oscar Alfredo Montenegro Arjona, Jairo Montenegro Arjona, Cristina Blasco Lafarga, Ana Cordellat

**Affiliations:** ^1^ Physical Education and Sports Studies Programme, ALTIUS Research Group, Universidad Surcolombiana, Neiva, Colombia; ^2^ Mathematics Department, Universidad Nacional de Colombia, Bogotá, Colombia; ^3^ Sport Performance and Physical Fitness Research Group (UIRFIDE), Physical Education and Sport Department, University of Valencia, Valencia, Spain

**Keywords:** high-intensity training, high-performance sports, lactate threshold training, endurance training, training load, training intensity distribution

## Introduction

In a recent paper, Treff et al. (2019) discussed and presented the polarization index (PI), which allows us to distinguish if a training intensity distribution (TID) of endurance athletes is polarized; this is achieved when PI > 2.

Succinctly, TID quantification is classified based on exercise distribution over time or distance into three zones comprising the “intensity zone-model”: Zone 1 (low intensity), Zone 2 (threshold intensity), and Zone 3 (high intensity). The total exercise prescription (100%) is distributed into these three zones (i.e., Zone 1 + Zone 2 + Zone 3 = 100%).

A polarized TID is defined based on two conditions: (*i*) a polarized structure, that is, Zone 1 > Zone 3 and Zone 3 > Zone 2 (in brief, Zone 2 < Zone 3 < Zone 1), and (*ii*) a relatively small proportion of Zone 2 (compared to zones 1 and 3).

## Discussion

We appreciate the establishment of PI and the cut-off >2 as an objective measure of how much intensity distribution there is in a polarized model that can be used by coaches.

This comment aims to point out some underlying conditions and analysis in the use of Formulas (1) and (2) proposed by Treff et al. (2019), and their graphical interpretation, apart from giving a more compact functional notation for those formulas that allow easy transcription into scientific software.

### About polarized TID conditions and the formulas

The three zones of an “intensity zone-model”, namely, Zone 1, Zone 2, and Zone 3, are denoted by *z*_1_, *z*_2_, and *z*_3_, respectively, and they are expressed in proportions instead of percentages. Regardless of units, this means that *z*_1_, *z*_2_, and *z*_3_ are real numbers in the interval [0,1] (i.e., 0 ≤ *z*_1_, *z*_2_, *z*_3_ ≤ 1) such that *z*_1_ + *z*_2_ + *z*_3_ = 1. The condition (*i*) for a polarized TID is 0 ≤ *z*_2_ < *z*_3_ < *z*_1_. The PI is made to capture the condition (*ii*).

This implies that PI calculation is only valid for TID that satisfies a polarized structure, that is, condition (*i*). Otherwise, we would be calculating the PI value of TID that does not meet condition (*i*), so it will never be a polarized TID independent of its PI.

From the aforementioned finding, if Zone 3 = 0, PI is zero per definition (Treff et al., 2019). In this case, we are dealing with TID that does not satisfy the polarized structure (condition (*i*)): it is not possible to have 0 ≤ *z*_2_ < 0 with some *z*_2_ in the interval [0,1]. However, the case *z*_3_ = 0 is not part of the cases, where we want to define PI.

Similarly, if Zone 3 > Zone 1, PI is not valid and must not be calculated (Treff et al., 2019); it is not remarkable (once we are dealing with TID that does not satisfy the polarized structure) since the case *z*_3_ > *z*_1_ belongs to the set of TID, for which PI is not defined, similar to the case *z*_2_ > *z*_1_ (taking into account that 0 ≤ *z*_1_, *z*_2_, *z*_3_ ≤ 1).

In Treff et al. (2019), some calculations are performed in addition to the PI analysis. In particular, with the aim of explaining that two different TIDs (e.g., *z*_1_ = 0.90, *z*_2_ = 0.05, and *z*_3_ = 0.05 and *z*_1_ = 0.74, *z*_2_ = 0.13, and *z*_3_ = 0.13) can give the same result, *PI* = 2. This example leads to ambiguity because it does not meet condition (*i*), which is known as a polarized structure (0 < *z*_2_ and *z*_2_ < *z*_3_, and *z*_3_ < *z*_1_). However, if we want to calculate PI for these values, we have

$$\log_{10}\left(100\left(\frac{0.90}{0.05}\right)\left(0.05\right)\right)=\log_{10}\left(90\right)\approx 1.9542$$

and

$$\log_{10}\left(100\left(\frac{0.74}{0.13}\right)\left(0.13\right)\right)=\log_{10}\left(74\right)\approx 1.8692.$$

These values are not equal to or close to 2.

### Calculation of the polarization index

Given the importance of using PI in sports training and in support of the proposal by Treff et al. (2019), the PI calculation can be reformulated compactly and clearly as a piecewise function. In this sense, we propose a modification to Eqs (1) and (2) proposed by Treff et al. (2019). Our function contemplates all the conditions by applying a single measure, excluding those TID that were not necessary to calculate.

Given a TID based on a three-zone model, with measurements *z*_1_, *z*_2_, and *z*_3_ of Zone 1, Zone 2, and Zone 3, such that *z*_1_, *z*_2_, *z*_3_ ∈ [0, 1] and *z*_1_ + *z*_2_ + *z*_3_ = 1, we define PI as follows:

$$\text{PI}=PI\left(z_{1},z_{2},z_{3}\right)=\left\{\begin{array}{cc} \log_{10}\left(100\left(\frac{z_{1}}{z_{2}}\right)\left(z_{3}\right)\right),\; & \text{if }0<z_{2}\text{ and }z_{2}<z_{3}\text{ and }z_{3}<z_{1}.\\ \log_{10}\left(100\left(\frac{z_{1}}{0.01}\right)\left(z_{3}-0.01\right)\right),\; & \text{if }z_{2}=0\text{ and }0.01<z_{3}\text{ and }z_{3}<z_{1}. \end{array}\right.$$ (1)

In other cases, PI is not defined.

The function *PI* defined in (1) makes explicit the adequate conditions on *z*_1_, *z*_2_, and *z*_3_ to obtain well-behaved Formulas (1) and (2), from Treff et al. (2019), on a more consistent and standard mathematical notation.

### Polarization index: a cut-off

Herein, we want to determine if TID, which satisfies the condition (*i*), is polarized. Treff et al. (2019) proposed a cut-off for PI of 2: if PI ≤ 2, TID is non-polarized. The reasoning to reach this cut-off is based on the analysis of their Figure 2, which shows us a plot in two dimensions that attempt to capture the interactions of three fractions of the intensity zones, but Figure 2 is hard to interpret to follow the arguments proposed therein.

**FIGURE 1:**
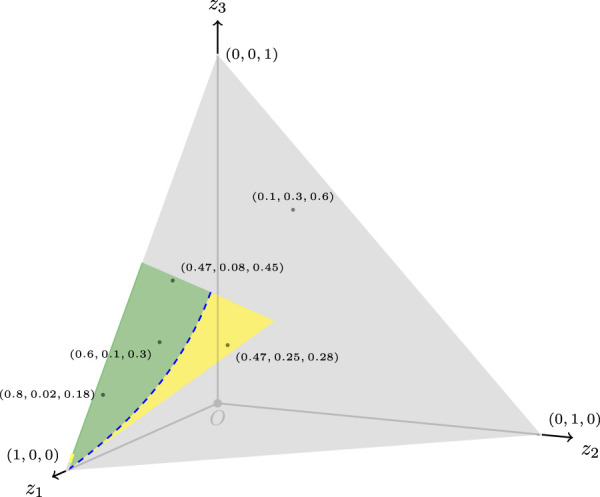
Domain of the PI function, a subset of a 3D space of points (*z*_1_, *z*_2_, *z*_3_), represented in green, blue, and yellow. These points satisfy condition (*i*). Green points represent *PI*(*z*_1_, *z*_2_, *z*_3_) > 2 (polarized TID), blue points (dashed line) represent *PI*(*z*_1_, *z*_2_, *z*_3_) = 2 (cut-off non-polarized TID), and yellow points represent *PI*(*z*_1_, *z*_2_, *z*_3_) < 2 (non-polarized TID). Gray points complete the set of points such that *z*_1_, *z*_2_, *z*_3_ ∈ [0, 1] and *z*_1_ + *z*_2_ + *z*_3_ = 1 (TID). Some points are tagged.

Taking into account that the function (1) proposed previously has its domain contained in a 3D space, that is, a set of points in the form (*z*_1_, *z*_2_, *z*_3_) that satisfy condition (*i*), a more accurate, easy to understand, and useful plot is shown in our Figure 1.

### Implementing the polarization index

An implementation example of function (1) in scientific software is the following snippet code written in R programming language (R Core Team, 2021) using a couple of functions from the package *dplyr*, part of *tidyverse* (Wickham et al., 2019). The function implementation verifies all the hypotheses for TID and condition (*i*).

require(dplyr)
f_PI <– function(z1, z2, z3){
 # First, verify the condition 0 <= z1, z2, z3 <=1 and z1 + z2 + z3 = 1
 if(0 <= z1 & z1 <= 1 & 0 <= z2 & z2 <= 1 &
   0 <= z3 & z3 <= 1 & dplyr::near(z1 + z2 + z3, 1)) {
  # PI Piecewise function verify condition (i)
  dplyr::case_when(
   0 < z2 & z2 < z3 & z3 < z1 ∼ log10(100 * (z1/z2) * (z3)),
   dplyr::near(z2, 0) & 0.01 < z3 & z3 < z1 ∼ log10(100 * (z1/0.01) * (z3-0.01)),
   TRUE ∼ as.double(NA)) # TID does not meet condition (i)
 } else {
  # It is not a TID
  as.double(NA)
 }
}

### Some example calculations

f_PI(0.6, 0.15, .25)
#> [1] 2
# *** Examples from Treff et al. (2019)

f_PI(0.6, 0.19, 0.21)
#> [1] 1.821617
f_PI(0.6, 0.14, 0.26)
#> [1] 2.046997
f_PI(0.8, 0.08, 0.12)
#> [1] 2.079181
f_PI(0.9, 0.05, 0.05)
#> [1] NA
f_PI(0.74, 0.13, 0.13)
#> [1] NA
# *** Some examples with z2 = 0
f_PI(1, 0, 0)
#> [1] NA
f_PI(0.99, 0, 0.01)
#> [1] NA
f_PI(0.9899, 0, 0.0101)
#> [1] -0.004408676
f_PI(0.9799, 0, 0.0201)
#> [1] 1.995503
f_PI(0.9701, 0, 0.0299)
#> [1] 2.165096
